# Community-based participatory-research through co-design: supporting collaboration from all sides of disability

**DOI:** 10.1186/s40900-024-00573-3

**Published:** 2024-05-10

**Authors:** Cloe Benz, Will Scott-Jeffs, K. A. McKercher, Mai Welsh, Richard Norman, Delia Hendrie, Matthew Locantro, Suzanne Robinson

**Affiliations:** 1 School of Population Health, Curtin University, Bentley, Australia; 2 Rocky Bay, Mosman Park, WA Australia; 3 Beyond Sticky Notes, Sydney, Australia; 4 Therapy Focus, Bentley, Australia; 5 Deakin Health Economics, Institute for Health Transformation, Deakin University, Melbourne, Australia

**Keywords:** Co-design, Community-based participatory-research, Telepractice, Disability, Lived experience, Method, Embedded researcher, Digital health, Patient and public involvement

## Abstract

**Background:**

As co-design and community-based participatory research gain traction in health and disability, the challenges and benefits of collaboratively conducting research need to be considered. Current literature supports using co-design to improve service quality and create more satisfactory services. However, while the *‘why’* of using co-design is well understood, there is limited literature on ‘*how*’ to co-design. We aimed to describe the application of co-design from start to finish within a specific case study and to reflect on the challenges and benefits created by specific process design choices.

**Methods:**

A telepractice re-design project has been a case study example of co-design. The co-design was co-facilitated by an embedded researcher and a peer researcher with lived experience of disability. Embedded in a Western Australian disability organisation, the co-design process included five workshops and a reflection session with a team of 10 lived experience and staff participants (referred to as co-designers) to produce a prototype telepractice model for testing.

**Results:**

The findings are divided into two components. The first describes the process design choices made throughout the co-design implementation case study. This is followed by a reflection on the benefits and challenges resulting from specific process design choices. The reflective process describes the co-designers’ perspective and the researcher’s and organisational experiences. Reflections of the co-designers include balancing idealism and realism, the value of small groups, ensuring accessibility and choice, and learning new skills and gaining new insights. The organisational and research-focused reflections included challenges between time for building relationships and the schedules of academic and organisational decision-making, the messiness of co-design juxtaposed with the processes of ethics applications, and the need for inclusive dissemination of findings.

**Conclusions:**

The authors advocate that co-design is a useful and outcome-generating methodology that proactively enables the inclusion of people with disability and service providers through community-based participatory research and action. Through our experiences, we recommend community-based participatory research, specifically co-design, to generate creative thinking and service design.

**Supplementary Information:**

The online version contains supplementary material available at 10.1186/s40900-024-00573-3.

## Plain language summary

Making better services with communities (called co-design) and doing research with communities (e.g. community-based participatory research) are ways to include people with lived experience in developing and improving the services they use. Academic evidence shows why co-design is valuable, and co-design is increasing in popularity. However, there needs to be more information on how to do co-design. This article describes the process of doing co-design to make telepractice better with a group of lived experience experts and staff at a disability organisation. The co-design process was co-facilitated by two researchers – one with a health background and one with lived experience of disability. Telepractice provides clinical services (such as physiotherapy or nursing) using video calls and other digital technology. The co-design team did five workshops and then reflected on the success of those workshops. Based on the groups’ feedback, the article describes what worked and what was hard according to the co-designers and from the perspective of the researchers and the disability organisation. Topics discussed include the challenge of balancing ideas with realistic expectations, the value of small groups, accessibility and choice opportunities and learning new skills and insights. The research and organisational topics include the need to take time and how that doesn’t fit neatly with academic and business schedules, how the messiness of co-design can clash with approval processes, and different ways of telling people about the project that are more inclusive than traditional research. The authors conclude that co-design and community-based participatory research go well together in including people with lived experience in re-designing services they use.

**Supplementary Information:**

The online version contains supplementary material available at 10.1186/s40900-024-00573-3.

## Introduction

Co-design has the potential to positively impact co-designers and their community, researchers, and organisations. Co-design is defined as designing with, not for, people [1] and can reinvigorate business-as-usual processes, leading to new ideas in industry, community and academia. As co-design and community-based participatory research gain traction, the challenges and benefits of collaborative research between people with lived experience and organisations must be considered [2].

Disability and healthcare providers previously made decisions for individuals as passive targets of an intervention [3]. By contrast, the involvement of consumers in their care [4] has been included as part of accreditation processes [4] and shown to improve outcomes and satisfaction. For research to sufficiently translate into practice, consumers and providers should be involved actively, not passively [4, 5].

Approaches such as community-based participatory research promote “a collaborative approach that equitably involves community members, organisational representatives and researchers in all aspects of the research process” [6] (page 1). This approach originated in public health research and claims to empower all participants to have a stake in project success, facilitating a more active integration of research into practice and decreasing the knowledge to practice gap^6^. Patient and public involvement (PPI) increases the probability that research focus, community priorities and clinical problems align, which is increasingly demanded by research funders and health systems [7].

As community-based participatory research is an overarching approach to conducting research, it requires a complementary method, such as co-production, to achieve its aims. Co-production has been attributed to the work of Ostrom et al. [8], with the term co-design falling under the co-production umbrella. However, co-design can be traced back to the participatory design movement [9]. The term co-production in the context of this article includes co-planning, co-discovery, co-design, co-delivery, and co-evaluation [10]. Within this framework, the concept of co-design delineates the collaborative process of discovery, creating, ideating and prototyping to design or redesign an output [11]. The four principles of co-design, as per McKercher [1], are sharing power, prioritising relationships, using participatory means and building capacity [1]. This specific method of co-design [1] has been used across multiple social and healthcare publications [10, 12–14].

A systematic review by Ramos et al. [15] describes the benefits of co-design in a community-based participatory-research approach, including improved quality and more satisfactory services. However, as identified by Rahman et al. [16], the ‘*why*’ is well known, but there is limited knowledge of ‘*how*’ to co-design. Multiple articles provide high-level descriptions of workshops or briefly mention the co-design process [13, 17–19]. Pearce et al. [5] include an in-depth table of activities across an entire co-creation process, however within each part i.e., co-design, limited descriptions were included. A recent publication by Marwaa et al. [20] provides an in-depth description of two workshops focused on product development, and Tariq et al. [21] provides details of the process of co-designing a research agenda. Davis et al. [11] discuss co-design workshop delivery strategies summarised across multiple studies without articulating the process from start to finish. Finally, Abimbola et al. [22] provided the most comprehensive description of a co-design process, including a timeline of events and activities; however, this project only involved clinical staff and did not include community-based participation.

As “We know the why, but we need to know the how-to” [16] (page 2), of co-design, our primary aim was to describe the application of co-design from start to finish within a specific case study. Our secondary aim was to reflect on the challenges and benefits created by specific process design choices and to provide recommendations for future applications of co-design.

## Overview of telepractice project

The case study, a telepractice redesign project, was based at Rocky Bay, a disability support service provider in Perth, Australia [23]. The project aimed to understand the strengths and pain points of telepractice within Rocky Bay. We expanded this to include telepractice in the wider Australian disability sector. The project also aimed to establish potential improvements to increase the uptake and sustainability of Rocky Bay’s telepractice service into the future. Rocky Bay predominantly serves people under the Australian National Disability Insurance Scheme (NDIS) [24] by providing a variety of services, including allied health (e.g. physiotherapy, dietetics, speech pathology, etc.), nursing care (including continence and wound care), behaviour support and support coordination [23]—Rocky Bay services metropolitan Perth and regional Western Australia [23].

The first author, CB, predominantly conducted this research through an embedded researcher model [25] between Curtin University and Rocky Bay. An embedded researcher has been defined as “those who work inside host organisations as members of staff while also maintaining an affiliation with an academic institution” [25] (page 1). They had some prior contextual understanding which stemmed from being a physiotherapist who had previously delivered telehealth in an acute health setting. A peer researcher, WSJ, with lived experience of disability, worked alongside CB. They had no previous experience in research or co-design, this was their first paid employment and they had an interest in digital technology. Peer Researcher is a broad term describing the inclusion of a priority group or social network member as part of the research team to enhance the depth of understanding of the communities to which they belong [26]. Including a peer researcher in the team promoted equity, collective ownership, and better framing of the research findings to assist with connecting with people with lived experience. These outcomes align with key components of community-based participatory research and co-design [27–30].

Person-first language was used as the preference of experts with lived experience who contributed to this research to respect and affirm their identity. However, we respect the right to choose and the potential for others to prefer identity-first language [31].

**Fig. 1:**
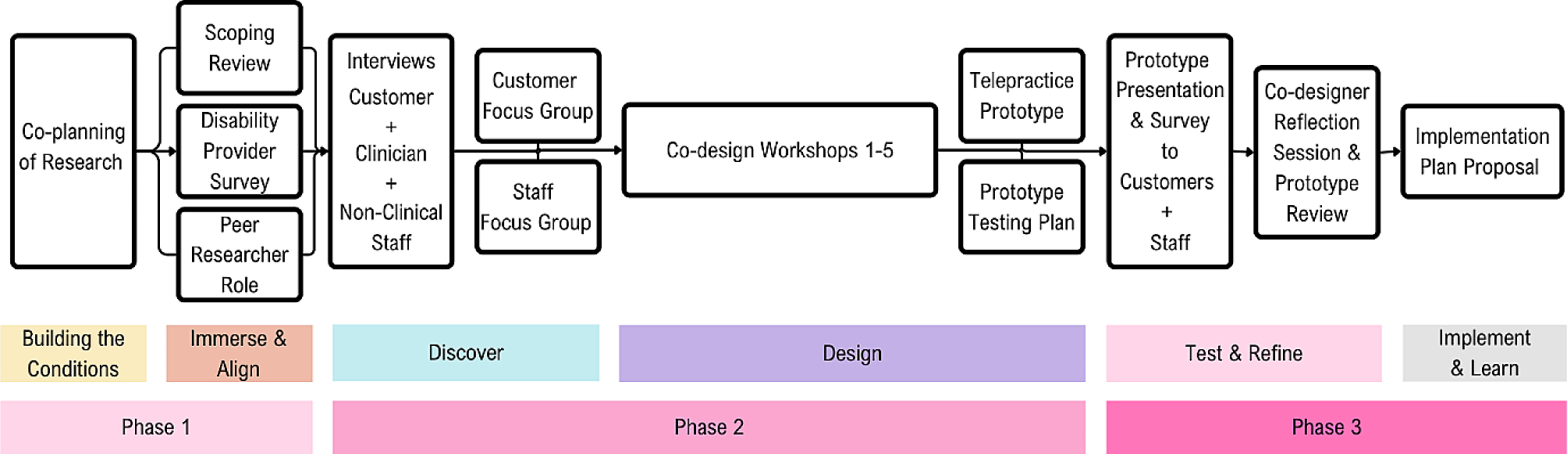
Summary of telepractice co-design project structure [1]

A summary of the structure of the phases completed before co-design workshops are represented in Fig. 1 below. Ethical approval for the project was received iteratively before each phase on the timeline (Fig. 1) from the Curtin Human Research Ethics Committee (HRE2021-0731). The reporting of this article has been completed in line with the Guidance for Reporting Involvement of Patients and the Public (GRIPP2) checklist [7].

Here, we present an outline of the chosen research methods with descriptions of each process design choice and supporting reasons and examples specific to the study. The format is in chronological order, with further details of each step provided in Appendix 1 (Supplementary Material 1).

## Methods and results

### Process of co-production and preparation for co-design

Co-production was chosen as the planning method for the study, as the inclusion of community members (Rocky Bay Lived experience experts and Staff) in each step of the research process would increase buy-in and make the research more likely to meet their needs [5]. An example of co-planning (part of co-production) includes the study steering committee, with a lived experience expert, clinician and project sponsor representatives collaborating on the selection of study aim, methods and recruitment processes. Another example of co-planning, co-design, and co-delivery was recruiting a peer researcher with disability, who worked with the embedded researcher throughout the study design and delivery.

The second process design choice was to attempt to build safe enough conditions for community participation, as people who feel unsafe or unwelcome are less likely to be able to participate fully in the research [1]. Building conditions for safety was applied by repeatedly acknowledging power imbalances, holding space for community input, and anticipating and offering accessibility adjustments without judgment.

### Getting started

Understanding and synthesising what is already known about telepractice experiences and learning from lived experience was prioritised as the first step in the process. We paired a scoping review of the literature with scoping the lived experiences of the community [32]. Our reasoning was to understand whether the findings aligned and, secondly, to learn what had already been done and to ask what was next, rather than starting from the beginning [1]. Examples of strategies used in this step included interviewing clinicians and service provider Managers across Australia to establish how they implemented telepractice during the pandemic and understand their views of what worked and what did not. The second learning process occurred onsite at Rocky Bay, with people with lived experience, clinicians and other support staff, whom the embedded researcher and peer researcher interviewed to understand experiences of telepractice at Rocky Bay.

The authors presented the interview findings during focus groups with Rocky Bay participants to share the learnings and confirm we had understood them correctly. The groups were divided into staff and lived experience cohorts, allowing for peer discussions and sharing of common experiences. This helped build relationships and a sense of familiarity moving into the workshop series.

### Co-design workshops

This section outlines specific components of the co-design workshop preparation before describing each of the five workshops and the final reflection session.

### Staff and community co-designers

Two process design choices were implemented to form the co-design group. The first was to prioritise lived experience input as there are generally fewer opportunities for lived experience leadership in service design [16], and because the disability community have demanded they be included where the focus impacts them [33]. To acknowledge the asymmetry of power between community members, people with lived experience of disability and professionals, we ensured the co-design group had at least the same number of lived experience experts as staff.

The second priority for the co-design group was to include people for whom involvement can be difficult to access (e.g. people who are isolated for health reasons and cannot attend in-person sessions, people who live in supported accommodation, part-time staff, and people navigating the dual-role of staff member while disclosing lived experience). It was important to learn from perspectives not commonly heard from and support equity of access for participants [4].

### Workshop series structure

When structuring the workshop series, lived experience co-designers nominated meeting times outside standard work hours to reduce the impact of co-design on work commitments and loss of income while participating. The workshops were designed to be delivered as a hybrid of in-person and online to give co-designers a choice on how they wanted to interact. The workshops were designed as a series of five sequential 90-minute workshops, where co-designers voted for the first workshop to be predominantly in-person and the remainder of the workshops online. Some co-designers chose to attend the initial session in person to build rapport. However, the virtual option remained available. The subsequent online sessions reduced the travel burden on co-designers, which the co-designers prioritised over further face-to-face meetings.

### Workshop facilitators

To maintain familiarity and ensure predictability for co-designers, the workshops were co-facilitated by the embedded researcher and peer researcher. The co-facilitators built on relationships formed through previous interactions (interviews and focus groups), and each facilitator represented part of the co-designer group as a clinician or a person with disability. An extra support person was tasked with supporting the co-designers with disability to break down tasks and increase the accessibility of activities. The reason for selecting the support person was that they could contribute their skills as a school teacher to support the communication and completion of activities, and they had no previous experience with disability services to influence the co-designers opinions. This role was adapted from the provocateur role described by McKercher [1].

## Pre-workshop preparations

To prepare for the workshops, each co-designer was asked to complete a brief survey to ensure the co-facilitators understood co-designers collect preferences and needs ahead of the session to enable preparation and make accommodations. The survey included pronouns, accessibility needs and refreshment preferences. Following the survey, the co-facilitators distributed a welcome video; the peer researcher, a familiar person, was videoed explaining what to expect, what not to expect and expected behaviours for the group to support a safe environment [1]. This process design choice was made to allow co-designers to alleviate any potential anxieties due to not having enough information and to increase predictability.

### Workshop resources and supports

As the first workshop was in-person, specific process choices were made to ensure co-designers felt welcome and to uphold the dignity of co-designers with lived experience [34]. Examples of process design choices include facilitating transport and parking requests, providing easy access to the building and room, making a sensory breakout room available and having the peer researcher waiting at the entrance to welcome and guide people to the workshop room.

After reaching the workshop room, all co-designers received an individualised resource pack to equalise access to workshop materials, aiming again to balance power in a non-discriminatory way [11]. The resource pack included name tags with pronouns, individualised refreshments, a fidget toy [35] whiteboard markers and a human bingo activity described in a later section. An easy-to-apply name tag design was selected after consulting a co-designer with an upper limb difference. Further details on the resource packs are included in Appendix 1 (Supplementary Material 1).

### Enabling different kinds of participation

**Fig. 2:**
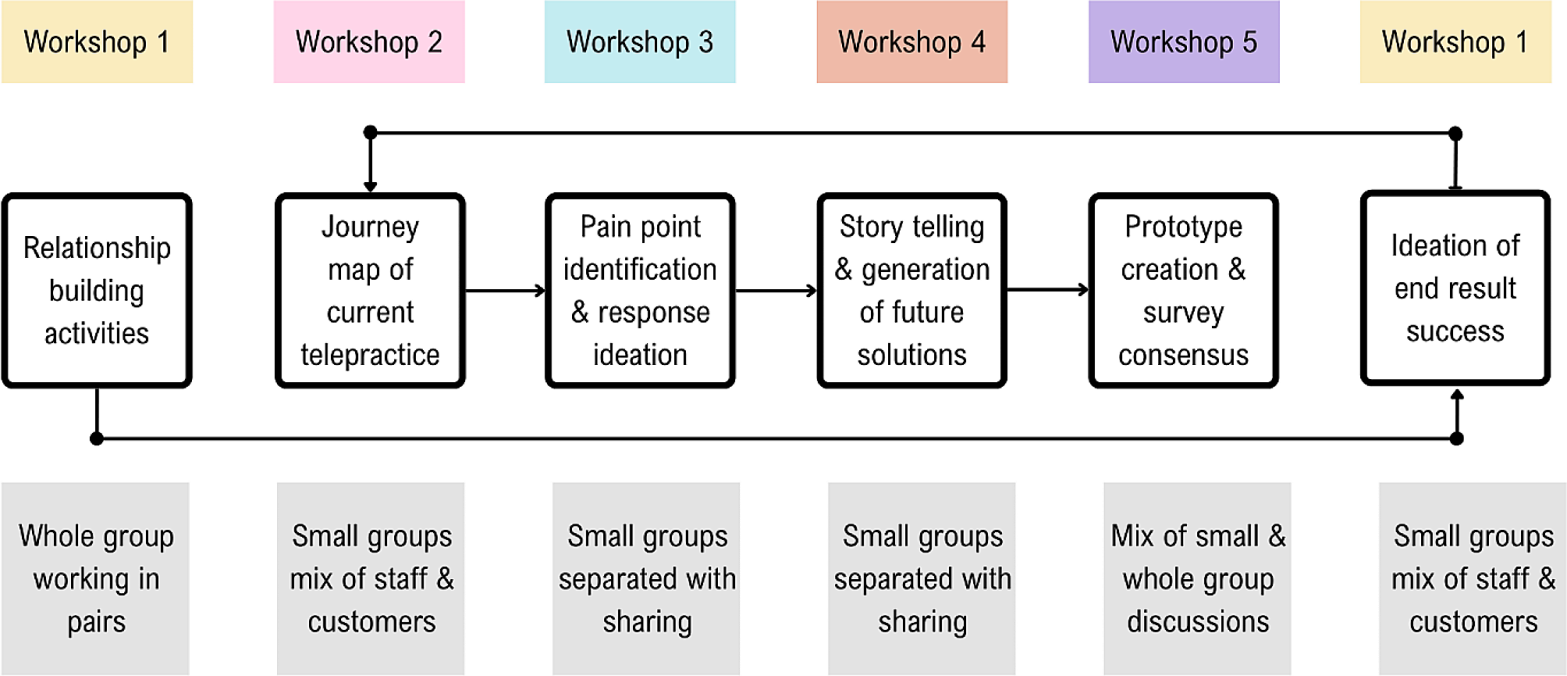
Non-verbal response cards

We provided non-verbal response cards to each co-designer as communication preferences vary significantly within the disability community. The cards were intended to benefit any co-designer who struggled to use the response buttons on MS teams. The co-facilitators co-created the Yes, No, and In-the-middle response cards (Fig. 2) and were guided by recommendations by Schwartz and Kramer [29]. They found that people with intellectual disability were more likely to respond “yes” if the negative option included a frowning face or red-coloured images, as choosing these types of alternatives was perceived as being negative or would cause offence [29].

**Fig. 3:**
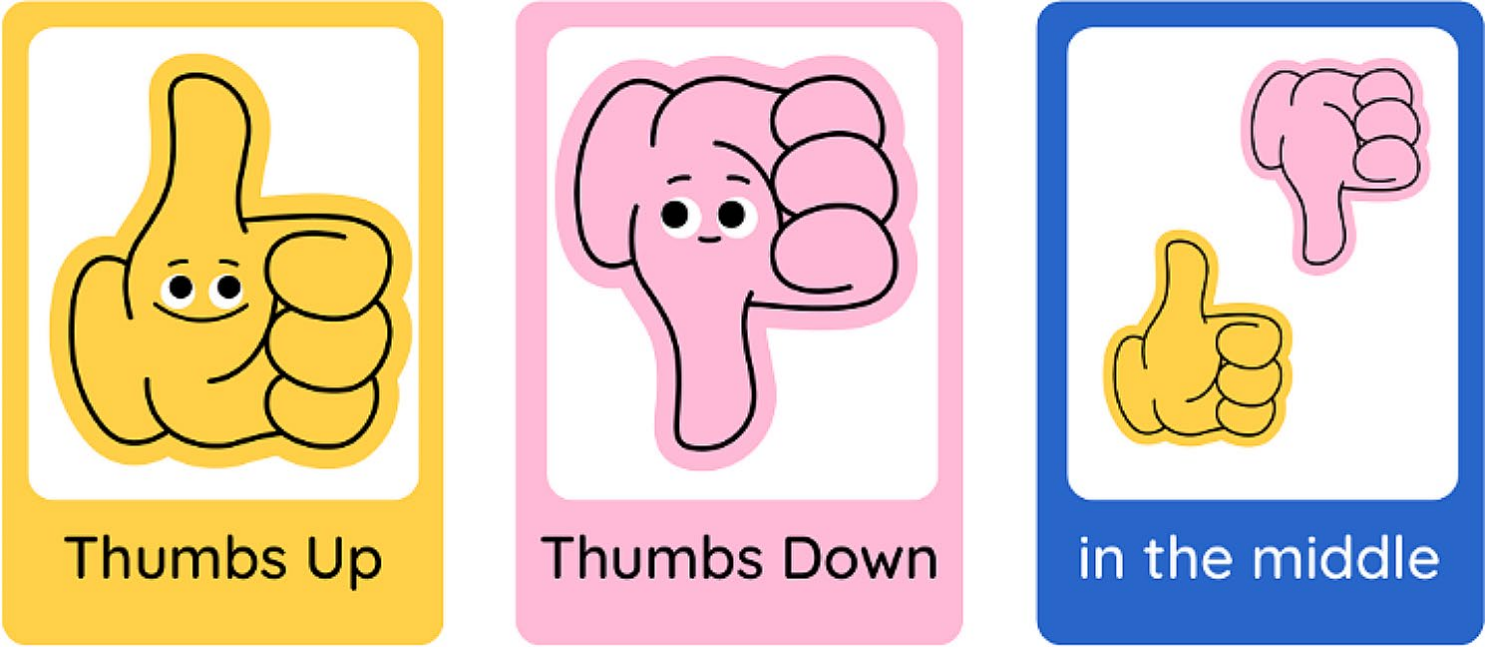
Outline of workshop and group structures

A summary of the structure and purpose of each of the five workshops is shown in Fig. 3, followed by a more in-depth discussion of the strategies employed in each workshop.

### Workshop 1: the beginning

Human Bingo was the first workshop activity, as it aimed to support relationship building in an inclusive way for both in-person and online attendees. The activity asked each co-designer to place a name in each worksheet box of someone who fit the described characteristic of that square(for example, someone who likes cooking). To include the two online attendees, laptops were set up with individual videocall streams and noise cancelling headphones enabling the online co-designers to interact one-on-one with others during the activities.

The second activity used *The Real Deal* cards by Peak Learning [36] to ask the co-designers to sort cards to prioritise the top five experiences and feelings they would want in a future version of telepractice. This activity aimed to set initial priorities for the redesign of telepractice [1]. Small groups with a mix of lived experience experts and staff were tasked with negotiating and collaborating to produce their top five desired experiences and feelings for future service success.

A follow-up email was sent after the session to thank co-designers, provide closure, invite feedback and let co-designers know what to expect from the next session.

### Workshop 2: mapping the journey

**Fig. 4:**
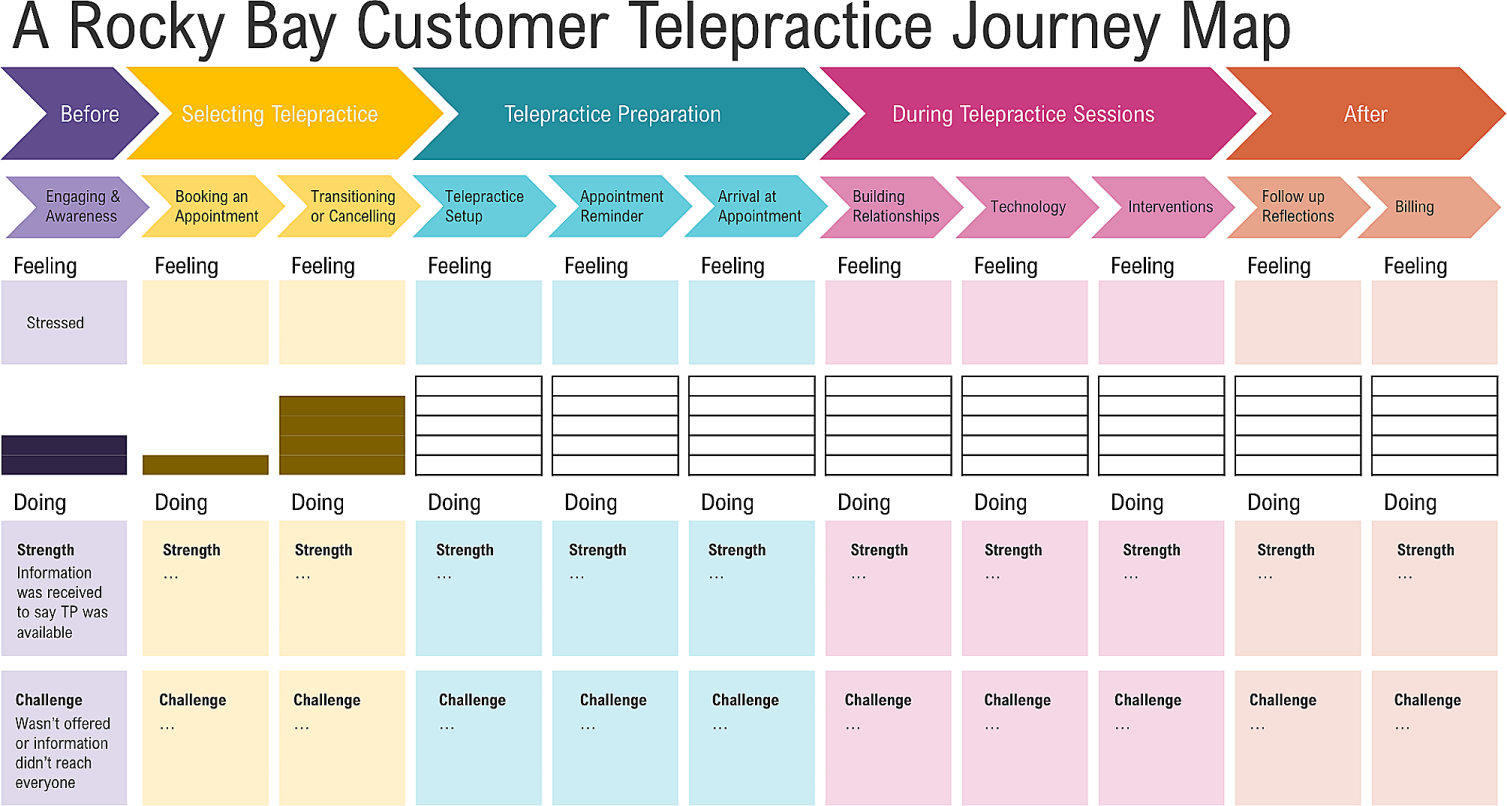
Draft journey map visualisation

In the second workshop, held online, the co-facilitators explained the journey mapping process and showed a draft of how the visual representation would likely look (Fig. 4). As the first step, co-designers were tasked with completing a series of activities to analyse lived experience interview data on the current experience of telepractice for lived experience experts. Small mixed groups were created, prioritising the needs of the lived experience experts to have staff who would be the best fit in supporting them to work through the task [1]. The small groups were allocated interview quotes corresponding to the steps of a customer journey through telepractice and asked to identify strengths, challenges and emotions associated with the current Telepractice service journey at Rocky Bay [1]. Further details on the journey map analysis are described in Appendix 1 (Supplementary Material 1) and in a published article co-authored by the co-designers (Benz et al. [37]).

After workshop two, the embedded researcher drafted a journey map by compiling the co-designer group responses to the analysis activity, which was then circulated for feedback and confirmation. The completed journey map is published with further details on the process in an article co-authored with the co-designers, Benz et al. [37].

### Workshop 3: ideas for addressing pain points

**Fig. 5:**
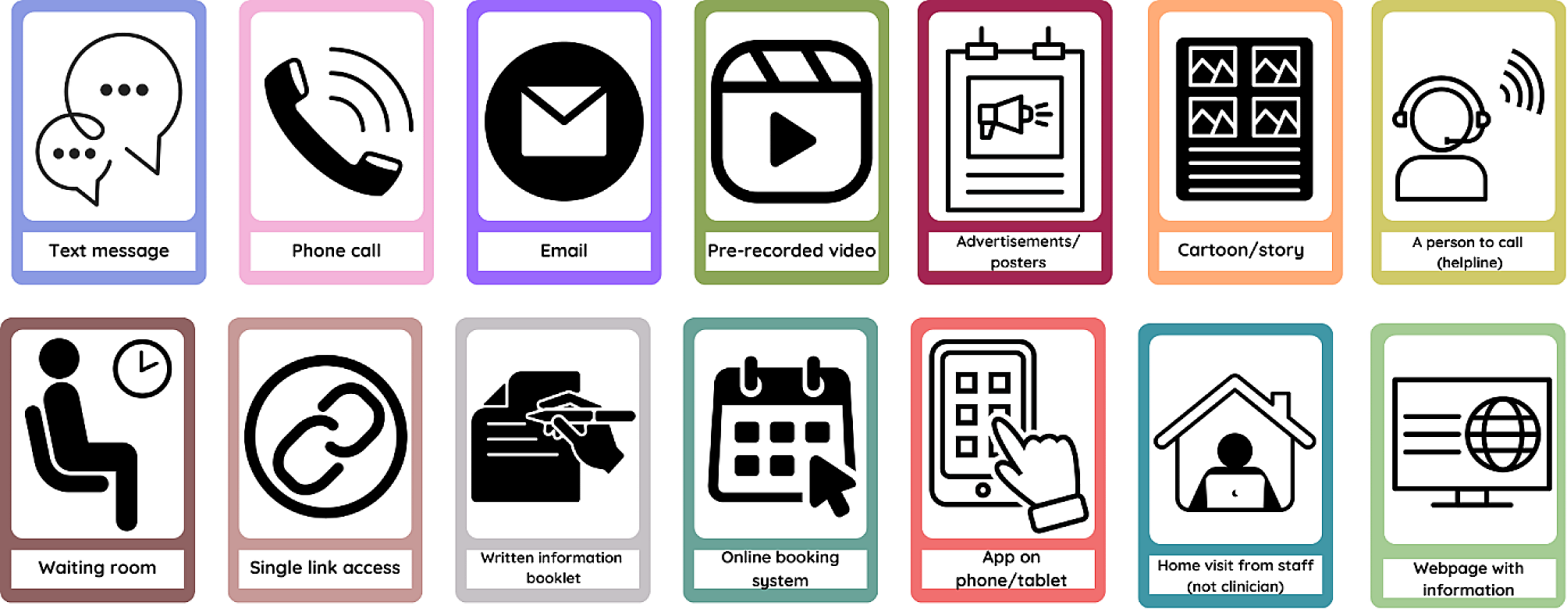
Option cards for Lived experience expert co-designer workshop activity

For the third workshop, the co-facilitators selected activities to be completed separately by lived experience and staff co-designers. The lived experience expert activity involved exploring preferences for improving pain points identified through the journey map. The lived experience expert activity was facilitated by the peer researcher and support person and included questions such as, *how would it be best to learn how to use telepractice?* Visual prompt cards were shared to support idea creation, where lived experience expert co-designers could choose any option or suggest an alternative (Fig. 5).

Simultaneously, the staff co-designers completed a parallel activity to address pain points from a service delivery point of view. These pain points were identified in the clinical and non-clinical staff interviews and from the journey map summary of lived experience expert interviews (analysed in Workshop 2). Staff co-designers completed a mind map based on service blueprinting guidelines by Flowers and Miller [38]. The activity used service blueprinting to identify a list of opportunities for improvement, with four prompts for co-designers to commence planning the actions required to implement these improvements. The foci of the four prompts were roles, policies, technology and value proposition [38] (described further in Appendix 1 (Supplementary Material 1)). Each of the four prompts were completed for the ten proposed opportunities for improvement to draft plans for future telepractice service delivery.

### Workshop 4: story telling and generation of future state solutions

In the fourth workshop, we introduced the concept of prototyping [39] as a designerly way to test co-designers’ ideas for improving telepractice according to desirability, feasibility and viability with a wider audience of lived experience experts and staff. The co-designers helped to plan the prototyping, and accessibility was a key consideration in selecting a prototype, as the group were conscious of the target audience.

Creating the prototype was collaborative, allowing co-designers to produce an output representing their ideas. They selected a video storyboard prototype with a staff and customer version formatted similarly to a children’s book. It included cartoon animations completed on PowerPoint, voiceover narration, closed captioning and an introductory explanation from two co-designers.

After workshop four, the co-designers collaborated on the customer and staff prototypes during the two weeks between workshops four and five, with support and input from the facilitators. The prototype files were co-produced, with different co-designers working on the visual aspects, the script for the main audio narration and the introductory explanation.

### Workshop 5: finishing the story

The co-design group reviewed the draft prototypes in the final workshop, with specific attention paid to the story’s cohesiveness.

The feedback questionnaire was then created to be completed by viewers outside of the co-design group after engaging with either the staff or the customer prototype. The survey allowed Rocky Bay customers and staff to contribute ideas. Following thoughtful discussions, consensus was reached by all co-designers on the final survey questions (Appendix 2 (Supplementary Material 1)).

A reflection activity concluded the final workshop, allowing co-designers to provide feedback on the co-design process, elements for improvement and aspects they valued in participating in the project. Their reflections on the benefits and challenges of co-design in this study are included in the section *Co-designer’s perspectives of the workshop series*, with the reflection questions included in Appendix 3 (Supplementary Material 1).

### Post prototype reflection session

The prototype feedback responses were reviewed with co-designers in a final reflection session. The group then discussed adaptations to the implementation plan for proposal to Rocky Bay. Following the survey discussion, co-designers reviewed proposed service principles for the new telepractice implementation recommendations. These principles aim to align any future decisions in the implementation and service provision stages of the telepractice project with the intentions of the co-designers. An additional reflection activity was completed, specific to the telepractice proposal they had produced and the prototyping process. Feedback relevant to subsequent discussions of the challenges and benefits of co-design is included in the following section: *Co-designer’s perspectives of the workshop series*, with the reflection prompts in Appendix 3 (Supplementary Material 1).

### Benefits and challenges

Learnings derived from completing a study of this kind are complex. However, it is necessary to reflect on which strategies used in the project were beneficial and which strategies created challenges - anticipated and unexpected. These reflections are discussed in two sections, the first being the challenges and benefits reflected upon by co-designers. The second set of reflections relates to organisational and research project-level benefits and challenges from the perspective of clinical department managers and researchers involved in the project.

### Co-designer’s perspectives of the workshop series

Co-designers were positive overall about the workshop series. Responses to a prompt for one-word descriptors of their experience included “captivating, innovative, fulfilling, exciting, insightful, helpful, eye-opening and informative*”*.

### Co-designing as a team

A foundational strategy implemented in this project was the intentional collaboration of lived experience experts with staff; this linked to the co-design principle of prioritising relationships and sharing power. Multiple reflections commented on feeling like a team and that having diverse perspectives across the group was beneficial.

*It was especially interesting to hear the perspective of clinicians (for us, the other side of Telepractice).* [Lived experience expert Co-designer]

Additionally, the combination of facilitators, including an embedded researcher with an allied health clinical background, a peer researcher with lived experience and a support person with strengths in breaking down tasks, provided different facets of support and task modelling to the co-designers throughout the process.

### Balancing idealism and realism

There is an inherent challenge in collaboration between lived experience experts and service providers, whereby co-designers formulate ideas for service improvement and then, in good faith, propose required changes to be implemented. Strategies to support imagination and idealism while being honest about the constraints of what can be delivered were implemented in the context of this project. This was essential to reinforce to co-designers that their contributions and ideas are valid while tempering their hopes with the truth that organisational change is challenging and funding for change is limited. Co-designers were encouraged to be cognisant of ideas that would require high investment (cost and time) and which ideas faced fewer barriers to implementation. This strategy did not prevent the ideation of changes and prioritising what mattered most to them, and co-designers felt it was beneficial in adding a level of consideration regarding what investments they deemed necessary versus those that would be nice to have. For example, having a person to call for help was viewed as necessary, while a nice to have was more advanced technological features.

*I feel that the prototype is useful; however, I worry that nothing will be carried over to the Rocky Bay Service. I feel like more customers will want to access telepractice, and Rocky Bay now needs to start the implementation process to ensure that telepractice is utilised, including processes, education and training.* [Clinician Co-designer]

#### The value of small groups

Working in small groups was another beneficial strategy, aiming to create a more hospitable environment for co-designers to voice their thoughts. The small groups varied across activities and workshops, with facilitators intentionally pairing groups that would best support the lived experience of expert co-designers completing activities. As described in the workshop sections, some activities suited mixed groups, whereas others suited lived experience expert and staff-specific groups. Two reflective comments demonstrated the benefit of the small groups, one from a clinician who reflected on supporting a fellow co-designer:

*I found that in our group, all of us had a say; however, [Lived Experience Co-designer name] was a bit overwhelmed at times, so I tried to support her with that.* [Clinician Co-designer]

And a lived experience expert co-designer additionally reflected:

*The breakout rooms were a very good idea. It can be quite intimidating speaking in front of the main group. I found it much easier to participate in the smaller groups*. [Lived experience expert Co-designer]

The second session included an unplanned whole group activity, which challenged co-designers. Co-designers reflections of this experience demonstrate the benefits of smaller groups:

*I did feel that at the end when the whole group did the task, there wasn’t as much collaboration as there were quite a few more assertive participants, so the quieter ones just sat back.* [Clinician Co-designer]

### Accessibility and choice

A challenge navigated throughout the workshop series with a diverse group of co-designers was meeting their varying individual health and other needs. This required responding in sensitive, non-judgemental, and supportive ways to encourage co-designers to engage fully. Examples of support include the presence of a support person and adaption of resource packs for co-designers who have difficulty swallowing (re: refreshments), as well as the previously mentioned non-verbal response cards and accessible name tags.

Accessibility supports were also provided for the peer researcher during facilitation activities, including pre-written scripts to provide clarity when explaining tasks to the co-design group, written reminders and regular check-ins. A lived experience expert co-designer reflected that it was beneficial that they could tell the peer researcher was nervous but appreciated that he was brave and made them feel like they did not need to be perfect if the peer researcher was willing to give it a go.

When facilitating the sessions, the embedded researcher and peer researcher identified that the workshops were long and, at times, mentally strenuous. One co-designer requested *“more breaks during each session”*. Breaks were offered frequently; however, upon reflection, we would schedule regular breaks to remove the need for co-designers to accept the need for a break in front of the group. The instructions for each activity were visual, verbal and written and given at the start of a task. However, once the co-designers were allocated to breakout rooms, they could no longer review the instructions. Many co-designers suggested that having the instructions in each breakout room’s chat window would have been a valuable visual reminder.

*One thing I think might of helped a little is having the instructions in the chat as I know I that I listened but couldn’t recall some of the instructions for the group task.* [Lived experience expert Co-designer]

#### Learning new skills and gaining new insight

The co-designers considered that the benefits of working together included learning new skills and widening their understanding of research, the services they provide or use, and the differences between the priorities of lived experience experts and staff. Two lived experience experts commented that the opportunity to learn collaboration skills and create cartoons using PowerPoint were valuable skills for them to utilise in the future. One clinician reflected that the process of co-design had improved their clinical practice and increased their use of telepractice:

*My practice is 100% better. I am more confident in using telepractice and more confident that, as a process, it doesn’t reduce the impact of the service- in some ways, it has enhanced it when customers are more relaxed in their own environments. I have not seen my stats, but my use of telepractice has increased significantly, too.* [Clinician Co-designer]

The management co-designer acknowledged that although ideas across the group may be similar, prioritisation of their importance can vary dramatically:

*Whilst all the feedback and potential improvements were very similar, some things that I viewed as not an issue, was very different to a customer’s perspective.* [Management Co-designer]

Overall, the workshop series challenged co-designers. However, the provision of a supportive and accessible environment resulted in mutual benefits for the research, organisation, and co-designers themselves. The strategy for facilitating the workshops was to pose challenges, support the co-designers in rising to meet them, and take into account their capabilities if provided with the right opportunity. A lived experience expert co-designer summarised the effectiveness of this strategy:

*I found the activities to be challenging without being too difficult. Each activity provided enough guidance and structure to encourage interesting group discussions and make collaboration easy.* [Lived experience expert Co-designer]

#### Research and organisational reflections of benefits and challenges of co-design

A significant challenge in completing this project was that building foundational relationships and trust takes time. While the authors view this trust as the foundation on which community-based participatory research and co-design are built, they note the direct tension of the time needed to develop these foundational relationships with the timeline expectations of academic and organisational decision-making. The flexibility required to deliver a person-centred research experience for the co-designers resulted in regular instances when timeline extensions were required to prioritise co-designer needs over efficiency. The result of prioritising co-designer needs over research timeline efficiency was an extended timeline that was significantly longer than expected, which sometimes created a disconnect between the flexibility of co-design and the rigidity in traditional academic and organisational processes.

The impacts of a longer-than-expected timeline for completion of the co-design process included financial, project scope, and sponsorship challenges. The project’s initial scope included a co-implementation and co-evaluation phase; however, due to the three-year time constraint, this was modified to conclude following the prototyping process. Whilst the three-year period set expectations for project sponsors and other collaborators from Rocky Bay, the wider context for the project varied significantly and rapidly over this period. This included two changes in Rocky Bay supervisor and one change in Rocky Bay project sponsor. Additionally, one of the academic supervisors left Curtin. This challenge indicates that the project would benefit from key role succession planning.

The peer researcher role was beneficial in providing an opportunity for a person with lived experience to join the study in a strength-based role and experience academic and business processes. However, challenges arose with the timeline extensions, which required this part-time, casual role to be extended by seven months. While the contract extension posed budgetary challenges, the role was viewed as vital to the completion of the project.

While an essential component of research, particularly involving vulnerable populations, ethical approvals proved challenging due to the non-traditional research methods involved in co-design. It was evident to the authors that while the ethics committee staff adhered to their processes, they were bound by a system that did not have adequate flexibility to work with newer research methods, such as co-design. Multiple methods in this study were heavily integrated into the community, including embedded research, peer research and co-design.

The present ethics process provided a comprehensive review focusing on planned interactions within research sessions (e.g. interviews and workshops). Unfortunately, this failed to account for a wider view, including the initial co-production prior to ethical application and anecdotal interactions that occurred regularly in the organic co-design process. In addition to the repeated submissions required to approve the sequential study format, these interactions created a significant workload for the research team and ethics office. These challenges were compounded by the need to navigate Rocky Bay’s organisational processes and changing business needs within ethical approval commitments.

In the authors’ opinion, prioritising the inclusion of lived experience experts in co-creating outputs to disseminate findings was beneficial. The co-creation enabled an authentic representation of the study to audiences regarding community-based participatory research and co-design method implementation. For example, the presentation of a panel discussion at a conference in which the peer researcher could prerecord his responses to questions as his preferred method of participation. All posters presented by the project were formatted to be accessible to lay consumers and were collaboratively produced, with the additional benefit of the posters being displayed across Rocky Bay hubs for customers and staff to gain study insights.

Due to the co-design method’s dynamic nature, some budgetary uncertainty was challenging to navigate. However, financial and non-financial remuneration for all non-staff participants in the project was prioritised. As previously discussed, the position of peer researcher was a paid role; additionally, all lived experience expert participants were remunerated at a rate of AUD 30/hour in the form of gift cards. The carer representative on the steering committee recommended using gift cards to avoid income declaration requirements from government benefits people may receive. Non-financial remuneration for the valuable time and contribution of the co-designer group included co-authorship on an article written regarding the Journey Map they produced (Benz et al. [37]) and acknowledgement in any other appropriate outputs. The implementation proposal provided to Rocky Bay included recommendations for continued inclusion and remuneration of co-designers.

#### Setting a new bar for inclusion

Another benefit to reflect upon, which may be the most significant legacy of the project, was setting the precedence for the inclusion of people with disability in decision-making roles in future projects and research conducted by the University and Rocky Bay. After this project commenced, other Rocky Bay clinical projects have similarly elevated the voices of lived experience in planning and conducting subsequent quality improvement initiatives.

*I’m lucky enough to have been part of a lot of projects. But I guess I probably haven’t been a part of continuous workshops, pulling in all perspectives of the organisation perfectly… So, collaboration and getting insight from others I haven’t usually was a very unique experience, and I definitely found value if this were to continue in other projects.* [Manager Co-designer]

## Discussion

In summary, the findings from using a co-design method for the telepractice research study produced a series of benefits and presented the researchers with multiple challenges. The findings also addressed a literature gap, presenting in-depth descriptive methods to demonstrate how co-design can be applied to a specific case.

Drawn from these findings, the authors identified six main points which form the basis of this discussion. These include (1) the fact that the necessary time and resources required to commit to co-design process completion adequately were underestimated at the outset, (2) there is a need to support the health, well-being and dignity of lived experience expert participants, (3) academic ethical processes have yet to adapt to address more participatory and integrated research methods, (4) strategies used to foster strong collaborative relationships across a diverse group were valued by all participants, (5) better delineation between terminologies such as co-design and community-based participatory research or patient and public involvement would improve the clarity of research methods and author intent and, (6) broader non-traditional impacts that participatory research can create should be better quantified and valued in the context of research impact. Each point will now be discussed in further detail.

In underestimating the time and resources required to complete the telepractice study, a scope reduction was required. This scope reduction removed the study’s originally planned co-implementation and co-evaluation phases. While Harrison et al. [40] and Bodden and Elliott [41] advocate for more frequent and comprehensive evaluation of co-designed initiatives, the authors acknowledge that this became no longer feasible within the study constraints. A growing body of literature indicates expected timelines for completed co-production projects from co-planning to co-evaluation. An example by Pearce et al. [5] indicated that a timeline of five years was reasonable. In contrast, a more limited co-design process was completed with a shorter timeline by Tindall et al. [13]. Although neither of these articles were published when this study commenced, they are complementary in building an evidence base for future research to anticipate an adequate timeline.

While co-design and other co-production processes are resource and time-intensive, the investment is essential to prioritise the health and other needs of potentially vulnerable population groups in the context of an imbalance of power [42]. In exploring the concept of dignity for people with disability, Chapman et al. [34] indicated that recognising the right to make decisions and proactively eliminating or minimising barriers to inclusion are key to protecting dignity. Community participation in decision-making processes such as this study can result in messy and unpredictable outcomes. However, the onus must be placed on policymakers, organisations, and academia to acknowledge this sufficiently rather than demand conformity [15].

The authors posit that the study would have benefited from an alternative ethics pathway, which may provide additional required flexibility while upholding the rigour of the ethical review process. The increasing frequency of participatory research studies indicates that challenges experienced by the authors of this study are unlikely to be isolated. Lloyd [43] described challenges regarding information gathered in-between, before and after structured research sessions, reflecting that they relied on personal judgement of the intent to consent for research use. Similarly, Rowley [44] reflected on the ethical complexities of interacting with families and respecting their confidentiality within the context of being integrated within an organisation. While these studies were co-production in child protection and education, the ethical challenges of their reflections parallel those experienced in the telepractice study. The risks posed by inadequate ethical support in these contexts are that increased poor ethical outcomes will occur, especially in the in-between times of co-design. Therefore, an ethics pathway that involves more frequent brief liaisons with a designated ethics representative to update project progress and troubleshoot ethical considerations may better support researchers to safeguard study participants.

We believe the decision to complete a sequential workshop series with a consistent group of diverse co-designers, led by co-facilitators, was a strength of the co-design process implemented in the telepractice re-design project. The group worked together across a series of workshops, which enabled them to build solid working relationships. Pearce et al. [5], Rahman et al. [16] and Tindall et al. [13] also demonstrated a collaborative whole-team approach to co-design. By contrast, studies that involved separate workshops with different cohorts or multiple of the same workshop did not demonstrate strong collaboration between co-designers [18–20]. Nesbitt et al. [19] explicitly highlighted that they would improve their method by completing sequential workshops with a continuous cohort. Stephens et al. [45] found that small mixed groups were not sufficient to support the participation of people with disability, indicating that the choice to intentionally balance groups to meet the lived experience expert co-designer’s needs may have been an impacting factor on our success.

A lack of clarity in the terminology used in co-design and community-based participatory practice was identified during the completion of this study. We found that co-design frequently meant either a collaborative design process or good participatory practices [46]. When viewing the structure of the telepractice re-design project, the overarching research approach was community-based participatory-research, and the method was co-design [9]. The delineation between the overarching approach and methods clarifies the misappropriation of the term co-design with the intent of meaning public participation [46] rather than the joint process of creative thinking and doing to design an output [11]. The use of the two-level structure appears more prominent in the United Kingdom, whereas Fox et al. [47] systematic review assessing public or patient participants identified that 60% of studies originated from the United Kingdom, compared to the next highest 16% for Canada or 4% from Australia and the United States. To improve clarity and reduce confusion about the terminology used, the authors advocate for greater awareness and implementation of the delineation between the concepts of a community-based-participatory-research/patient or public involvement approach versus the co-design method.

An example of co-design being used where alternate terms such as community-based participatory processes (or research) may be more relevant was the most recent amendment to the act governing the NDIS under which this project resided [48]. The term co-design could be interpreted as an intent to collaborate with people with disability for equitable involvement in all aspects of the NDIS [48]. It is proposed that the differentiation of these terms would assist in clarifying the intent of the study and dissuade inaccurate expectations of community involvement or design processes.

Implementing community-based participatory research has demonstrated the potential to create an impact that expands further than the original aim of the study. The skills learned by co-designers, the learning of the research team in collaboration with people with disability, the engagement and skill-building of a peer researcher with lived experience, the organisations who engaged in the co-design process and the academic and lay people who engaged with research outputs, all carry a piece of the impact of the co-design process. Rahman et al. [16] contend that co-design processes positively impact communities. In the context of this study, the peer researcher was included in the National Disability Insurance Agency’s quarterly report as an example of strength-based employment opportunities, which significantly positively impacted his career prospects [49]. This project provided skills for people with disability that they value and improved the clinical practice of clinician co-designers, which echoes the conclusions of Ramos et al. [15], who described that participants felt valued and experienced improved self-esteem. There is additional intent from the authors to positively impact disability providers and academia, to advocate for greater collaboration, and to provide open-access publications to provide a stronger evidence base for co-design in clinical practice and service delivery.

### Strengths and limitations

The study provides reflective evidence to support the challenges and benefits experienced during the implementation of the study. However, a limitation in the project’s design was the exclusion of outcome measures to assess the impact of process design choices directly. Stephens et al. [45] completed targeted outcome measures correlating to accessibility adaptations in co-design and conceded that the variability of findings and individual needs reduced the usefulness of these measures.

The reduction of project scope enabled the completion of the study within the limitations of budgeting and timeline restrictions. Although the scope of the project had some flexibility, there were limitations to how far this could be extended as resources were not infinite, and staffing changes meant that organisational priorities changed. Including implementation and evaluation would have improved the study’s rigour. However, Rocky Bay now has the opportunity to implement internally without potential research delays and restrictions.

The blended and flexible approach to the co-design process was a strength of the study as it met the co-designers needs and maximised the project’s potential inclusivity. This strength has the potential to positively impact other studies that can modify some of the process design choices to suit their context and increase inclusivity [11]. It is believed that the messiness of co-design is important in meeting the needs and context of each individual study; therefore, no two co-design processes should look the same.

The authors concede that the inclusion of a cohort of people with disability and clinical staff does not represent the entirety of their communities, and their proposed changes may cause some parts of the disability community to experience increased barriers [50]. It is important to note that while the co-designers who participated in this project provided initial design developments, future opportunities remain to iterate the proposed telepractice service and continue to advocate for equitable access for all.

## Recommendations for future studies

Recommendations from this study fall into two categories: recommendations for those intending to utilise the described methods and recommendations for future avenues of research inquiry. For those intending to implement the methods, the primary recommendations are to build ample time buffers into the project schedule, implement key role succession planning and set remuneration agreements at the outset, and work together as partners with the mindset that all contributors are creative [51] with important expertise and invaluable insights if supported appropriately.

Regarding avenues for future inquiry, we recommend investigating a more dynamic and flexible ethics process that may utilise more frequent short consultations to respond to ethical considerations during the emergent co-design and participatory research.

## Conclusion

In the authors’ opinion, supported by co-designers experiences, co-design is a useful and outcome-generating methodology that can proactively enable the inclusion of people with disability and service providers in a community-based participatory research approach. The process is both time and resource-intensive; however, in our opinion, the investment is justified through the delivery of direct research benefits and indirect wider community benefits. We advocate for using community-based participatory-research/processes paired with co-design to generate creative thinking within service design processes. Through co-design processes, we recommend collaborating with a single diverse group of co-designers who have the time and space to build trusting working relationships that enable outputs representative of the group consensus.

### Electronic supplementary material

Below is the link to the electronic supplementary material.

**Supplementary Material 1:** Appendix 1–3

## Data Availability

The dataset supporting the conclusions of this article is predominantly included within the article (and its additional files). However, due to the small number of co-designers reflecting upon the research, despite deidentification, there is a reasonable assumption of identification; therefore, the reflection activity response supporting data is not available.

## Abbreviations

AUD: Australian Dollar
GRIPP2: Guidance for Reporting Involvement of Patients and the Public 2 Checklist
HREC: Human Research Ethics Committee
PhD: Doctor of Philosophy
PPI: Patient and Public Involvement
MS Teams: Microsoft Teams
NDIS: National Disability Insurance Scheme
