# Supplementary material for: Community-based participatory-research through co-design: supporting collaboration from all sides of disability
Source: Res Involv Engagem. 2024 May 10;10:47. doi: 10.1186/s40900-024-00573-3 (PMC11084036; doi:10.1186/s40900-024-00573-3)
Supplement: Supplementary file 1 — Supplementary Material 1: Appendix 1–3 [file 40900_2024_573_MOESM1_ESM.docx]

**Appendix 1:**

**Process of co-production and preparation for co-design**

The process of co-production is multi-faceted and requires careful and considered planning to support including the right people in the right ways, with trust etc. While the design component of the process is the predominant focus of the case study presented below, a brief overview of the completed preparatory steps will be outlined first. The iterative process of building the conditions^1^ for a successful co-design process is essential, this process continues throughout the entirety of the project to ensure a safe and welcoming environment for co-designers (people with disability, Rocky Bay staff etc.). From the outset, co-production was built in from conception to completion, including an oversight steering committee with customer representation, working with a peer researcher, co-planning activities, co-authoring outputs, the researcher being embedded in the organisation and working iteratively with the co-designers throughout the design process.

**Getting Started:**

The initial phase of the project sought to understand the needs of Rocky Bay and other similar organisations across Australia to establish a baseline and potential areas of improvement. This wide-lens exploration was followed by a narrow-lens investigation of telepractice related experiences of customers, clinicians and non-clinical support staff at Rocky Bay. Each participant of the study completed a one-on-one interview with either the peer researcher (for customers) or the embedded researcher (for staff), followed by the opportunity to attend customer- or staff-specific focus groups to reflect on the interview findings. Following the focus groups, participants were informed about design workshops to be conducted in the next step of the co-design process, and that the research team would contact them individually to discuss their potential participation.

**Co-design Workshops**

This section breaks down the co-design process by group, structure, facilitation and finally described each of the five workshops.

**Staff and community co-designers**

The project participants were offered the opportunity to express their interest to participate in a co-design workshop series conducted in a hybrid of in-person (session 1) and virtual (session 2-5) formats due to travel burden, increased risk of virus transmission and stress of clinical environments exclusively in-person sessions were not preferred by people with disability. Of those who expressed their interest, the selection of the co-designers aimed to have at least the same number of customers than of staff^1^ acknowledging the asymmetry of power. The final co-design group included ten members, five Rocky Bay staff and five customers, one of which had become a staff member in the administrative team of the clinical department in the time between their initial interview and the commencement of the workshops. Within the context of the workshops this co-designer was given the choice, when relevant, to participate in the customer or staff groups.

The customer group included three wheelchair users, a person with low vision and hearing loss, two people with upper limb impairment and one person who chose to attend virtually due to high risk of contracting respiratory viruses. The staff co-designers included four clinicians across occupational therapy, dietetics, speech pathology and nursing and the final co-designer was the management representative responsible for briefing senior management, business case writing and implementing the outputs of co-design.

**Workshop Series Structure:**

The co-design workshops were conducted as a set of five sequential 90-minute workshops with the same group of co-designers, in a hybrid of in-person (workshop 1) and online (workshops 2-5) delivery. Through consultation with the customer co-designers, a mutually accepted location for the initial session was selected. For timing a Tuesday after hours’ timeslot was selected to facilitate customer co-designers attending after work to decrease disruptions and improve accessibility^1^.

**Workshop Facilitators:**

The embedded researcher (CB) and peer researcher (WSJ) facilitated the workshops with the support of an additional support person Katie Harris (KH), as it was identified that a support person assist with breaking down tasks, asking questions to clarify instructions and advocate for the needs of the customer co-designers, similar to the provocateur role described by McKercher^1^. However, it was recognised that any person with experience in supporting people with disability, could have personal experience interacting with disability services or providers which may influence their interaction with the workshop content. Therefore, with appropriate ethical approvals, a primary school teacher known to the first author was approached to help, as their experience in supporting the completion of classroom activities was viewed as suitable for the role.

**Pre-Workshop Preparations:**

In the weeks prior to the workshops, each attendee was asked to complete a brief survey including their preferred name and pronouns, their preference for beverage or snack, and any travel or other support needs to assist them in attending the workshops in-person or virtually. They were also provided with a workshop-specific participant information statement and consent form, which provided additional information to the original information and consent provided prior to the initial interview and focus groups. One week prior to the first workshop, each attendee was sent a short workshop information video recorded and captioned by the peer researcher describing what to expect from the workshops, what not to expect and what the expected code of behaviour to support a safe working environment for all co-designers^1^.

**Workshop Resources and Supports**

The first workshop focused on the need for the co-designers to build relationships with each other and familiarise themselves with the group, redesign context, facilitators and types of tasks used throughout the workshop series. As the first workshop was to be held in-person, special focus was put on meeting the needs of the customer attendees in terms of transport, parking, access to the building and room and having sufficient space to comfortably move around, inclusive of any wheelchair users. A location was selected, with the peer researcher calling each of the customer co-designers to confirm the location and assess any transport support requirements, while the embedded researcher liaised with the staff attendees to confirm their ability to attend. As a strategy recommended by McKercher^1^ to decrease stress or discomfort of arriving co-designers, the peer researcher was waiting at the entry of the location to welcome everyone and direct them to the correct room where the first author was waiting to provide information regarding rest room facilities and a sensory break room provided for those with sensory accessibility needs.

Using the information gathered through the pre-workshop questions, each co-designer was provided on arrival with a care package providing all required resources to ensure equitable workshop participation^3^. A name tag was included with their chosen name and pronouns, and confirmation was sought from a co-designer with upper limb impairment as to the easiest nametags to wear. All staff co-designers were asked to wear neutral non-work attire to the workshops and no other name badges or identifiers to limit any power imbalances that may be derived from professional appearances or attire^1^. Other items included were an Acknowledgement of Country and code of care card^1^ describing the behaviour principles of the workshops and a human bingo activity card which was used for an activity described below. In addition to the resource cards, each co-designer’s individualised care package included their beverage choice, snack preference, a sensory fidget toy^5^, a whiteboard marker and wipes, and a set of sticky note tabs.

**Enabling different kinds of participation**

Co-designers were given a set of response cards to enable non-verbal and visual responses to questions if this was the communication preference of the participant. The peer researcher (WJS) and the embedded researcher (CB) developed the tool together Figure 3 below. Consideration of the contents of each card were guided by the insights from Schwartz and Kramer^4^ which worked with a group of peer researchers in creating a participant reported outcome measure for young people with intellectual disabilities. Findings of this article included the emphasis that people with intellectual disability were more likely to respond yes if the alternative had frowning face or red coloured images as they were perceived as being negative and to avoid offence^4^. In deference to these findings, all cards were designed to include smiling faces and no cards were coloured red or green to minimise any subconscious biases towards the green/good response versus the red/bad response card, as shown in Figure 3.


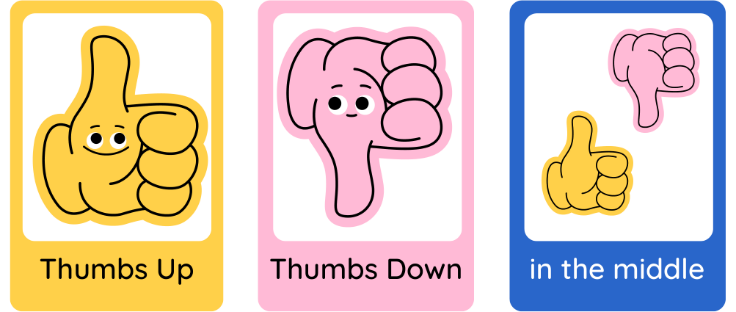


**Figure 3: Non-verbal response cards** (half page width)

**Workshop 1: The Beginning**

The first activity of the workshop was a human bingo card which required placing the name of a co-designer in each box who fit the described characteristic of that square as an opportunity to start building relationships. As a strategy to accommodate the two co-designers unable to attend in-person, two laptops were set up with individual videocall streams enabling them to interact one-on-one with other co-designers during the activities through noise cancelling headphones and listen with the group to instructions. The workshop facilitators participated in this activity alongside the co-designers as it was deemed important for the relationship between facilitators and co-designers to build in congruence with the group relationships.

The second activity used sets of *The Real Deal* cards by Peak Learning^6^ to facilitate an activity that asked the co-designers to sort cards to prioritise the top five experiences and feelings they would want in a future version of telepractice. The co-designers were split into four small groups with a mix of customers and staff, and each group was tasked with negotiating and collaborating to produce their top five experiences and feelings desired for future service success. This task was intended to assist co-designers to understand what they were aspiring to achieve with the co-design process^1^.

**After Workshop 1**

Following workshop one a summary of the session was sent to each co-designer that included the opportunity to provide feedback and a reminder of the time and place of the next session.

**Workshop 2: Mapping the Journey**

The second workshop was held via Microsoft Teams (MS teams) and aimed to produce a journey map of the current customer experience of telepractice across five phases including (1) before, (2) selecting telepractice, (3) telepractice preparation, (4) doing telepractice sessions and (5) after. In preparation for this workshop, first author CB analysed the customer responses from the initial one-on-one interviews and tentatively placed excerpt quotes along a timeline representing the journey customers take through telepractice delivered services. With assistance from peer researcher WSJ these excerpts were compiled into PowerPoint presentations, each presentation including one of the five phases across the timeline and colour-coded to match that section on the proposed journey map visualisation.

The co-designers were allocated into four groups with one member of each group consenting to sharing their screen in a breakout room for their group. This co-designer was sent one of the files from phases 2-5 to work through prior to the session in preparation. Following a brief introduction and recap, the facilitators described the journey mapping process and showed draft visual. The first phase was used as an example demonstration by the facilitator CB, and co-designers were able to ask questions prior to moving into breakout rooms. Each group was comprised of a mixture of customer and staff members, prioritising the needs of the customers to have staff who would be the best fit in supporting them to feel comfortable and safe working through the task^1^.

Each group worked collaboratively to complete the exercises outlined in their presentation slides from the data excerpts corresponding to their allocated phase of the journey map timeline. Following the completion of the task, the groups returned to the central room to describe their findings to the remainder of the co-designers. One component of the activity was accidently skipped by a group and was subsequently completed by the whole co-design group with the facilitators at the end of the session.

**After Workshop 2**

A draft of the journey map was circulated for feedback from co-designers following the compilation of answers by first author CB into the visualisation. The process of developing the journey map and the resultant visualisation is described in further depth elsewhere with co-authorship of the co-designers.

**Workshop 3: Ideas for Patching the Pain Points**

Co-designers reviewed and confirmed the journey map at the beginning of the third workshop. Following this, the group split into two groups, with the customers completing an activity that established their preferences for how some of the challenges identified in the journey map may be remedied. Simultaneously, the staff members of the co-design group were completing a parallel activity to address pain points from a service delivery point of view. These pain points were identified in the clinical and non-clinical staff interviews as well as from the journey map summary of customer interviews. The groups were split to make room for divergence of needs and perspectives and mitigate any perceived power over solutions not directly impacting them.

The customer activity was supported by WSJ (peer researcher) and KH (educator facilitator) and included the group responding to seven questions:

1. How would it be best to access information re: telepractice and spread awareness?
2. How would it be best to understand when and why it might (or might not) be good to choose telepractice?
3. How would it be best to learn about how to use telepractice?
4. How would it be best to access support for set up / troubleshooting?
5. How would it be best to be reminded of sessions and updates?
6. How would it be best to choose between delivery modes?
7. How would it be best to improve virtual experience? (tech features)

A visual prompt card was shared on the screen to support ideation, from which customers could choose any option to answer the question or propose an alternate option. The prompt card supported customer co-designers to analyse current available and feasible options while simultaneously building a platform for them to link to additional ideas.

Staff in workshop three completed a mind map which was first draft by first author CB through a process based on the blueprinting guidelines of Flowers and Miller^7^. The activity uses current state service blueprinting to identify a concrete list of opportunities for improvement, derived from interview data and the customer journey map, with four prompts for co-designers to commence planning the actions required to implement these improvements. The four prompts were:

1. Roles: which role and responsibilities would encompass this proposed improvement and who would need to be involved in the development and implementation of this action?
2. Policies: which policies or guidelines currently in place may need to be amended, or what new policy or guideline may need to be created in the development and implementation of this action?
3. Technology: what systems and tools currently exist within the organisation to enable this action, or what systems or tools may need to be acquired?
4. Value Proposition matrix: measuring the expected level of effort and level of value of completing each of the proposed opportunities for improvement.

Each of the four prompts were completed for the ten proposed opportunities for improvement to form the initial ideation for the future state of telepractice from a service delivery perspective.

**Workshop 4: Story Telling and Generation of Future State Solutions**

The fourth workshop commenced by reflecting on the activities completed thus far and introduced the idea of prototyping^8^ as a method of displaying the proposed ideas and testing their desirability, feasibility and viability with a wider audience of customers and staff? The co-designers helped to plan the prototyping by progressing through the steps of deciding what to test, deciding what they were trying to learn and who they wanted to learn it from, establishing what roles people would take on to complete the prototype, and selecting a type of prototype and method of testing it. Each of these steps was completed as a group using a shared presentation which included options for categories of prototypes available, and how those options could be tested. accessibility was a key consideration in the selection of a prototype as the group were conscious of creating a prototype that would be viewed by people with disability. An additional goal for the group was to choose a prototype that they could virtually collaborate on from different locations. The co-designers selected a storyboard with separate customer and staff-focused versions in a picture book format which could be created in shared PowerPoint files, narrated, and presented in a video by the co-designers. The method of testing was to be a short online survey in which viewers of the prototype storybook video could click a link or scan a QR code to complete, allowing them to give their thoughts and suggestions for improvements.

After co-planning the prototype, the co-designers were given a summary of the information gathered in the previous workshops and split into four groups, made up of either customers or staff to commence work on creating stories to describe potential future telepractice experiences. These stories were sent to the facilitators at the conclusion of the workshop to commence drafting the prototypes.

**After Workshop 4**

Co-production of the customer and staff prototype versions were collaborated on by the co-designers during the two-week period between workshops four and five, with support and input from the facilitators. The prototype files were co-produced, with different co-designers working on the visual aspects, the script for the main audio narration and introductory explanation.

**Workshop 5: Finishing the Story**

At the final co-design workshop the draft prototypes were reviewed and iterated by each of the small groups. Once each group had a chance to review their stories, the full drafts were reviewed for comment by the whole co-design group, with specific attention paid to the cohesiveness of the story presented in the prototypes.

The final activity for the prototype review was to discuss what feedback questions to ask viewers after engaging with either the staff or customer prototype. The survey format included a customer and staff stream; however, the questions were in principle the same, with wording variations to accommodate the different audiences. Following thoughtful discussions, consensus was reached by all co-designers on the final survey questions (Appendix 1).

The conclusion of the final workshop gave co-designers an opportunity to provide their feedback on the co-design process, elements for improvement and aspects they valued in participating in the project. Their reflections are included in the section *Co-designer’s perspectives of the workshop series*,reflecting on the benefits and challenges of the way co-design was implemented.

**Post Prototype Reflection Session**

A final reflection session was held with co-designers to review the findings of the prototype testing, discuss adaptations to the current plan and input on the implementation plan to be proposed to Rocky Bay. The findings of the survey from prototype testing were presented to the co-designers as a one-page visual infographic, with an option to access a long format summary.

Following discussion of the results of the survey and proposed changes to the telepractice design, the co-designers reviewed a set of proposed service principles for the new telepractice implementation, which seek to align any future decisions in the implementation and service provision stages to the intentions of the co-design team. The final aspect of the session was for co-designers to reflect on their thoughts and feelings of the proposal for telepractice re-design which they had produced and gained feedback on through prototyping. Feedback relevant to subsequent discussions of challenges and benefits of co-design are included in the following section *Co-designer’s perspectives of the workshop series*.

## References

1. McKercher KA. Beyond Sticky Notes Doing co-design for Real: Mindsets, Methods, and Movements. 1 ed. Sydney, NSW: Beyond Sticky Notes; 2020. 225 p.

2. Staniszewska S, Brett J, Simera I, Seers K, Mockford C, Goodlad S, et al. GRIPP2 reporting checklists: tools to improve reporting of patient and public involvement in research. Research Involvement and Engagement. 2017;3(1):1-11. doi:10.1186/s40900-017-0062-2

3. Davis A, Gwilt I, Wallace N, Langley J. Low-Contact Co-Design: Considering more flexible spatiotemporal models for the co-design workshop. Strategic Design Research Journal. 2021;14(1):124-137. doi:10.4013/sdrj.2021.141.11

4. Schwartz AE, Kramer JM. Inclusive approaches to developing content valid patient‐reported outcome measure response scales for youth with intellectual/developmental disabilities. British Journal Learning Disability. 2021;49(1):100-110. doi:10.1111/bld.12346

5. Flattery S. Stim Joy: Using Multi-Sensory Design to Foster Better Understanding of the Autistic Experience: ProQuest Dissertations Publishing; 2023

6. Peak Learning. The Real Deal [Internet]. 2023 [cited 6.10.2023]. Available from: https://www.peaklearning.com/trd/

7. Flowers E, Miller ME. Your Guide to Blueprinting The Practical Way. 1 ed. USA: Practical By Design 2022. 134 p. p. 1-134.

8. Blomkvist J. Benefits of Service Level Prototyping. The Design journal. 2016;19(4):545-564. doi:10.1080/14606925.2016.1177292

**Appendix 2:**

Prototype Survey Questions

b

**Appendix 3:**

Workshop 5 Reflection Activity

We have are a series of questions to help you with reflecting on this experience, you are welcome to answer all of them or any of them. You are welcome to provide your feedback in any format you wish, including written, verbal/audio recorded, visual drawings/art etc. We will start the conversation today and then you can send through your final thoughts when you’re ready via email (or post if you’d like to). We would love to include some of your thoughts in the published work of this project eg. The submission to rocky bay and my PhD and you can let us know if you’d like your name to not be included with your feedback, and of course we will show you what would be included before we do it.

The questions are:

1. How easy have the activities been to do?
2. How much have you felt a part of the team in this process?
3. Is there any key parts of the experience you’d like to share with us?
4. Why do you think this work matters?
5. What might you like to be different?
6. What did you love and learned in this process?
7. **What was the biggest change from being a part of this project?**

Reflection Session Activity

These questions were provided via a qualtrix survey with a QR code and link during the reflection session with co-designers:

1. What do you think about the Telepractice prototype/proposed service that has been created?
2. If you could describe your involvement the new telepractice service design in one word, what would it be?
3. What is the most important thing for this project to achieve? (if only one thing could be guaranteed what would it be?)
4. Any final thoughts or comments?
